# Upper and Lower Limb Movement Kinematics in Aging *FMR1* Gene Premutation Carriers

**DOI:** 10.3390/brainsci11010013

**Published:** 2020-12-24

**Authors:** Zheng Wang, Callie Lane, Matthew Terza, Pravin Khemani, Su Lui, Walker S. McKinney, Matthew W. Mosconi

**Affiliations:** 1 Department of Occupational Therapy, University of Florida, Gainesville, FL 32611-0164, USA; zheng.wang@ufl.edu; 2 Kansas Center for Autism Research and Training (K−CART) and Life Span Institute, University of Kansas, Lawrence, KS 66045, USA; 3 Department of Physical Therapy and Rehabilitation Science, University of Kansas Medical Center, Kansas City, KS 66160, USA; clane5@kumc.edu; 4 Department of Applied Physiology and Kinesiology, University of Florida, Gainesville, FL 32611-8205, USA; MJT023@ufl.edu; 5 Department of Neurology, Swedish Neuroscience Institute, Seattle, WA 98121, USA; Pravin.Khemani@Swedish.org; 6 Huaxi Magnetic Resonance Research Center (HMRRC), Department of Radiology, West China Hospital of Sichuan University, Chengdu 610041, China; lvsu@wchscu.cn; 7 Clinical Child Psychology Program, University of Kansas, Lawrence, KS 66045, USA

**Keywords:** *FMR1* gene, fragile X−associated tremor/ataxia syndrome, gait, reaching, kinematics, cerebellum, basal ganglia

## Abstract

Fragile X-associated tremor/ataxia syndrome (FXTAS) is a neurodegenerative disorder associated with a premutation cytosine-guanine-guanine (CGG) trinucleotide repeat expansion of the *FMR1* gene. FXTAS is estimated to be the most common single-gene form of ataxia in the aging population. Gait ataxia and intention tremor are the primary behavioral symptoms of FXTAS, though clinical evaluation of these symptoms often is subjective, contributing to difficulties in reliably differentiating individuals with FXTAS and asymptomatic premutation carriers. This study aimed to clarify the extent to which quantitative measures of gait and upper limb kinematics may serve as biobehavioral markers of FXTAS degeneration. Nineteen premutation carriers (aged 46–77 years), including 9 with possible, probable, or definite FXTAS and 16 sex- and IQ-matched healthy controls, completed tests of non-constrained walking and reaching while both standing (static reaching) and walking (dynamic reaching) to quantify gait and upper limb control, respectively. For the non-constrained walking task, participants wore reflective markers and walked at their preferred speed on a walkway. During the static reaching task, participants reached and lifted boxes of different sizes while standing. During the dynamic reaching task, participants walked to reach and lift the boxes. Movement kinematics were examined in relation to clinical ratings of neuromotor impairments and CGG repeat length. During non-constrained walking, individuals with FXTAS showed decreased stride lengths and stride velocities, increased percentages of double support time, and increased variabilities of cadence and center of mass relative to both asymptomatic premutation carriers and controls. While individuals with FXTAS did not show any static reaching differences relative to the other two groups, they showed multiple differences during dynamic reaching trials, including reduced maximum reaching velocity, prolonged acceleration time, and jerkier movement of the shoulder, elbow, and hand. Gait differences during non-constrained walking were associated with more severe clinically rated posture and gait symptoms. Reduced maximum reaching velocity and increased jerkiness during dynamic reaching were each related to more severe clinically rated kinetic dysfunction and overall neuromotor symptoms in *FMR1* premutation carriers. Our findings suggest kinematic alterations consistent with gait ataxia and upper limb bradykinesia are each selectively present in individuals with FXTAS, but not asymptomatic aging premutation carriers. Consistent with neuropathological and magnetic resonance imaging (MRI) studies of FXTAS, these findings implicate cerebellar and basal ganglia degeneration associated with neuromotor decline. Our results showing associations between quantitative kinematic differences in FXTAS and clinical ratings suggest that objective assessments of gait and reaching behaviors may serve as critical and reliable targets for detecting FXTAS risk and monitoring progression.

## 1. Background

Fragile X-associated tremor/ataxia syndrome (FXTAS) is estimated to be the most common single-gene form of ataxia in the aging population [1,2]. It is caused by premutation alleles consisting of 55–200 cytosine-guanine-guanine (CGG) trinucleotide repeats in the 5′ untranslated region of the *FMR1* gene [3,4]. Approximately 45% of male and 16% of female *FMR1* premutation carriers over 50 years develop FXTAS. The primary symptoms include gait ataxia, intention tremor, and Parkinsonism, though symptom presentation is variable across individuals, and some patients also show cognitive decline, psychiatric issues, and autonomic dysfunction, among other comorbidities [1,2,5,6]. Multiple radiological signs also inform diagnosis, including hyperintensities of the middle cerebellar peduncle seen on T2-weighted magnetic resonance imaging (MRI), generalized cerebral atrophy, and white matter hyperintensities of the splenium of the corpus callosum [1,2,7,8]. Due to the heterogeneous clinical and neuroanatomical presentations of FXTAS, the disease mechanisms are not yet well understood. As such, many patients initially are misdiagnosed or diagnosed only after significant progression [9]. Objective approaches for reliably identifying FXTAS are needed to advance our understanding of the disease mechanisms and guide differential diagnosis and patient tracking.

Sensorimotor behaviors represent promising targets for clarifying mechanisms of FXTAS and defining new biomarkers because (1) sensorimotor deficits are core defining features of FXTAS [1,2]; (2) they are highly quantifiable in both the spatial (e.g., movement amplitude) and temporal (e.g., movement duration, velocity, and acceleration) domains, allowing precise characterization of subtle changes; and (3) they are supported by neocortical, striatal, and cerebellar brain networks implicated in both MRI and postmortem studies of FXTAS [8,10,11].

Despite offering promise as targets for understanding disease mechanisms, sensorimotor issues have been the subject of a relatively small number of quantitative studies of FXTAS. Previous sensorimotor studies of FXTAS have demonstrated atypicalities in precision manual motor control [12,13], postural control [14,15,16], and step initiation [8,13], suggesting that multiple sensorimotor behaviors are compromised in carriers with FXTAS. More subtle sensorimotor issues also have been documented among asymptomatic premutation carriers, including postural and precision manual control issues [12,14,17]. In particular, female carriers without FXTAS have shown subtle gross motor deficits (e.g., gait and step initiation) relative to asymptomatic male carriers during dual-task activities that demand working memory processing [18,19]. These findings suggest that sensorimotor deficits may manifest in aging *FMR1* premutation carriers and differentially affect females prior to the onset of FXTAS. Quantitative strategies to measure these subtle sensorimotor changes hold promise for identifying subclinical traits associated with aging in premutation carriers prior to clinically observable disease symptoms. Quantitative studies are needed to determine the extent to which different sensorimotor issues are specific to FXTAS and which may develop early during aging in premutation carriers who are pre- or asymptomatic.

Gait and upper limb movements may be especially important targets for understanding FXTAS and establishing new disease-specific quantitative markers, because gait ataxia and intention tremor are (1) major clinical signs of FXTAS [1,2], (2) included in standard neurological evaluations of FXTAS [20], and (3) critical for adaptive daily living skills and quality of life. During daily activities, lower limbs support postural stability and mobility [21,22], while upper limb control is necessary for manipulating objects [23,24]. When examined in combination, gait and reaching movements are planned in a coordinative and compensatory manner [25]. Furthermore, the majority of daily living skills involve the coordination of both lower and upper limb movement (e.g., carrying a glass of water from one place to another). Both upper and lower limb deficits have been documented in studies of aging *FMR1* premutation carriers, but simultaneous analysis of upper and lower limb movement features has not been done, and evaluating how they are related provides a more ecologically valid assessment of the motor functions in premutation carriers and may provide a more sensitive approach for detecting degeneration relative to the measurement of either upper or lower limb movements in isolation.

Using the instrumented Timed Up and Go (i-TUG) test, O′Keefe et al. previously indicated that individuals with FXTAS show a decreased stride amplitude, increased stride variability, and prolonged transition between different movements relative to healthy controls and age-matched premutation carriers without FXTAS [17]. Notably, gait issues also were associated with age, cognitive decline, and increased CCG repeat length in carriers with FXTAS [18,19,26,27], suggesting that quantitative gait assessment may be useful for identifying FXTAS severity and risk. While the i-TUG test has provided valuable knowledge about performance−based mobility, understanding is limited regarding locomotor control in *FMR1* premutation carriers with and without FXTAS during non-constrained walking and activities involving the upper limbs. This knowledge gap is important because non-constrained walking, assessed using a kinematic motion capture system, shows high levels of test–retest reliability compared with performance-based gait tests, including the i-TUG test, suggesting that it may be particularly useful for tracking disease progression or prodromal degeneration [26]. Furthermore, lower limb-facilitated reaching, including reaching-in-standing (i.e., static condition) and walking-for-reaching (i.e., dynamic condition), is critical for multiple daily activities (e.g., walking to pick up a glass from a dining table), suggesting that they may relate strongly to functional abilities and their decline.

Kinematic studies of the gait and reaching behaviors may also provide important insights into neural systems affected in FXTAS. For example, the cerebellum has been repeatedly implicated in FXTAS, and studies of reaching in patients with cerebellar ataxia have documented target overshooting [28,29], reduced coordination of multijoint movements [30], and reduced end point accuracy [31,32]. Consistent with the hypothesis that cerebellar degeneration plays a prominent role in sensorimotor issues associated with FXTAS, we recently showed that increased variability of precision manual motor behavior is associated with reduced functional connectivity between cerebellar Crus I and the extrastriate cortex in aging premutation carriers [10]. To our knowledge, no studies have simultaneously examined the gait and reaching in aging *FMR1* premutation carriers with and without FXTAS.

The current study had two objectives: (1) quantify disease-related behavioral patterns by examining locomotor and upper limb kinematics during tasks of non-constrained walking and arm reaching in healthy controls and premutation carriers with and without FXTAS, and (2) determine the relationships of the gait and reaching kinematic variables with disease risk factors, including age, CGG repeat length, cognitive ability (i.e., IQ), and the neurological functions associated with FXTAS. For the first objective, we quantified individuals’ non-constrained gaits across spatial, temporal, and kinematics dimensions, as our group had previously identified deficits in these dimensions from other sensorimotor activities in *FMR1* premutation carriers [10,12,13,14]. Based on previous studies of walking in *FMR1* premutation carriers [17,26,27,33], we hypothesized that individuals with FXTAS would show a reduced stride length, decreased stride velocity, and increased percentage of double support time (i.e., percent of stride time spent with both feet in contact with the ground), as well as increased variability of stride length and cadence relative to healthy controls and asymptomatic carriers. As musculoskeletal weakness has been consistently documented in individuals with FXTAS [1,34,35,36,37], we also predicted that patients would show reduced knee flexion of the swing leg at toe-offs and reduced knee extension and ankle dorsiflexion of the ipsilateral leg at heel strikes. For tests of reaching, we hypothesized that premutation carriers would show jerkier joint movement relative to healthy controls, reflective of reduced multi-joint coordination that is often observed in cerebellar patients [30,38,39,40]. We also examined reaching duration, maximum reaching velocity, and acceleration time, as they represent common targets in studies of intention tremor and Parkinsonism, two symptoms commonly seen in FXTAS. We also hypothesized that reaching deficits would be more severe during dynamic trials of walking-for-reaching, suggesting that gait impairments interrupt upper limb performance during goal-oriented activities [25,41]. For the second objective, we hypothesized that increased abnormalities of the gait and reaching kinematics in *FMR1* premutation carriers would be associated with increased age, CGG repeat length, and clinically rated movement issues, as well as lower IQ scores, as they are all factors contributing to FXTAS risk, severity, and progression [1,2].

## 2. Materials and Methods

### 2.1. Participants

Nineteen *FMR1* premutation carriers and 16 controls matched by age and sex were enrolled in this study. *FMR1* premutation carriers were identified through our fragile X clinics and postings on local and national fragile X association listservs. Healthy controls were recruited through community advertisements. Individuals were excluded if they reported lower extremity orthopedic surgery within the past year, any musculoskeletal disorder associated with an atypical gait, or a history of medications known to affect sensorimotor functioning [42]. No individual reported any neurological concerns (e.g., postural and gait instability, tremor, or lack of coordination) during the screening interview. *FMR1* premutation carriers completed genetic testing to quantify the CGG repeat length, and a structured neurological evaluation using the International Cooperative Ataxia Rating Scale (ICARS) [43], conducted by a movement disorder specialist (P.K.). All participants completed a T2-weighted MRI and an abbreviated battery of the Stanford–Binet Intelligence Scales, Fifth Edition (SB-5) [44] to quantify cognitive abilities.

*FMR1* premutation carriers were classified as FXTAS+ (*n* = 9), FXTAS− (*n* = 6), or inconclusive (*n =* 4) based on diagnostic standards [1]. The FXTAS+ group included individuals meeting the diagnostic standards for definite (*n* = 1), probable (*n* = 6), or possible FXTAS (*n* = 2). The FXTAS− group included premutation carriers with no radiological or clinical signs of FXTAS (*n* = 6). Four premutation carriers who failed to complete the neurological evaluation due to scheduling issues (*n* = 3) or the MRI scan due to claustrophobia (*n* = 1) were categorized as inconclusive and were excluded from the analyses of the FXTAS+, FXTAS−, and control groups (i.e., the first objective of the study). Eight participants (5 premutation carriers and 3 healthy controls) reported being on medication within 48 h of testing, including antidepressants (selective serotonin reuptake inhibitors: 2 premutation carriers and 1 control; other antidepressants: 3 premutation carriers), sedatives or hypnotics (1 premutation carrier), levothyroxine (2 controls), or a mood stabilizer (1 premutation carrier).

### 2.2. Procedures and Approaches

All procedures involved in this research were approved by the institutional review board at the UT Southwestern Medical Center and Children’s Hospital of Dallas in accordance with the Declaration of Helsinki. The IRB number is STU 052013-43 with an approval date of 13 August 2013. Written consent was obtained from each adult individual before the administration of tests and procedures.

#### 2.2.1. T2-Weighted Magnetic Resonance Imaging (MRI) Scan

All participants completed a T2-weighted MRI scan (repetition time = 6350 ms; echo time = 100 ms; flip angle = 120°; field of view = 256 × 156 × 256 mm^3^; 78 axial slices; voxel size = 1 mm^2^ × 2 mm; no gap) to evaluate radiological abnormalities associated with FXTAS [1,7,45]. T2-weighted scans were analyzed by a trained radiologist (S.L.) with expertise in neurological disorders.

#### 2.2.2. CGG Repeat Length

The CGG repeat length was examined for all premutation carriers. Molecular testing was conducted at Dr. Berry-Kravis’ Molecular Diagnostic Laboratory at Rush University. Genomic DNA was isolated from peripheral blood leukocyte samples. The *FMR1* polymerase chain reaction test with quantification of the allele-specific CGG repeat length was performed using commercially available kits (Asuragen, Inc., Austin, TX, USA).

#### 2.2.3. Neurological Examination

*FMR1* premutation carriers completed a structured neurological evaluation, including the ICARS, administered by a movement disorder specialist with expertise in ataxia (P.K.). The ICARS is comprised of 19 sections, examining postural and gait disturbances, kinetic function (i.e., limb ataxia), dysarthria and speech disorders, and oculomotor issues. Higher scores indicate more severe neuromotor issues [46,47].

#### 2.2.4. Gait Assessment

All sensorimotor assessments were administered on an AMTI AccuGait walkway (American Mechanical Technology, Inc., Watertown, MA; length: 2.9 m, width: 1.2 m). Prior to testing, 42 passive reflective markers were attached to major joints, based on an industry standardized Plug-in-Gait Full Body template (Vicon, Centennial, CO, USA). Kinematic data were recorded using ten Vicon Vantage V5 cameras and a motion capture system with a spatial error of 2 mm and a sampling rate of 100 Hz.

A starting and a finishing line were set 2 m away from the entrance and exit of the AccuGait walkway for a total of 6.9 m of walking distance for each individual. Participants started each trial by standing upright at the starting line with their feet side-by-side. After receiving an audio cue, participants began walking continuously toward the finish line at their preferred speed. All participants completed three trials of non-constrained walking while barefoot. Practice trials were administered prior to data recording to ensure participants felt comfortable wearing the reflective markers and understood task instructions.

To limit variability in the rates at which individuals increased or decreased their walking speed at the beginning and end of the walkway, only kinematic data from the middle of the walkway representing individuals’ preferred speeds were analyzed. The heel strikes and toe-offs of each leg were manually labeled on the raw kinematic data by a trained scorer and double-checked by a separate trained scorer (M.T.). Kinematic data were then exported, filtered, and analyzed using custom scripts in Matlab 2019A (MathWorks, Inc., Natick, MA, USA). A low-pass fourth order Butterworth filter was applied at a cutoff frequency of 7 Hz to filter the raw kinematic data.

Consistent with prior studies examining gait impairments across the spatial, temporal, and kinematic domains in other clinical populations [48,49], dependent gait variables were derived from the post-processed kinematic time series and separated into these three categories. The spatial features of the gaits included stride length (anterior–posterior distance from the point-of-heel strike of one leg to the point-of-heel strike of the same leg) and step width (lateral distance between the heel markers of the leading and trailing legs at the heel strike of the leading leg). Temporal features of the gaits included stride duration (duration between two consecutive heel strikes of the same leg), stride velocity (ratio of stride length over stride time), percentage of double support time (percent of stride time spent in the double-limb support phase for the leading leg), and cadence (number of steps per minute). Kinematic features included the standard deviation of the center of mass (COM) in the mediolateral direction and the ankle and knee joint angles of the ipsilateral and contralateral legs at heel strikes and toe-offs. The gait variables of the left and right leg were pooled to derive each individual’s mean and coefficient of variation (CoV) across legs and strides. The CoV was calculated as the standard deviation/mean to provide information on gait variability while accounting for differences in mean performance.

#### 2.2.5. Upper Limb Assessment

**Figure 1:**
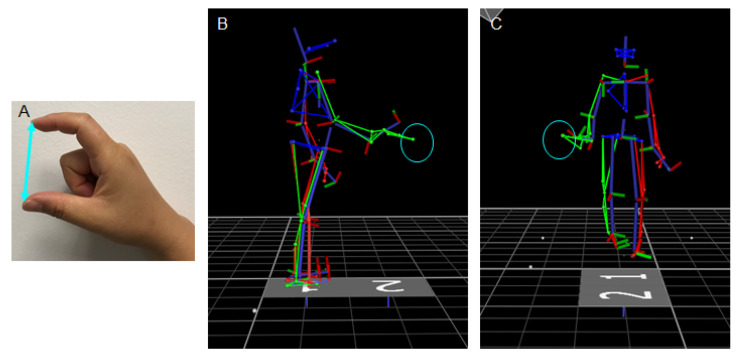
(**A**) A participant used her index finger and thumb to form a C-shaped pose to allow her hand aperture to be determined. The examiner measured the distance (cyan line) between the finger tips as the length of the participant’s hand aperture. (**B**) A skeleton figure, depicted in the sagittal plane to show a representative reaching while standing trial when the participant reached a target box (cyan circle). The target was set at one arm’s length from the participant in alignment with the midline of his body to allow him to bend forward to reach and lift the target. (**C**) A skeleton figure depicted in the frontal plane to show a representative walking-for-reaching trial prior to the participant reaching the target box (cyan circle). The participant reached the box with his trunk facing forward and the right arm slightly extended to the right.

To assess the upper limb movement characteristics of *FMR1* premutation carriers, tests of static and dynamic reaching were administered. Prior to the tests, individuals’ hand apertures were measured to ensure the size of the target boxes was individualized. Participants were requested to form a C-shaped pose using the right thumb and index finger (Figure 1A), and the measurement was used as a reference to select from eleven toy boxes with edge lengths of 1.5, 3.6, 5.0, 5.8, 7.0, 8.4, 9.2, 10.5, 11.4, 12.4, or 13.4 cm. Boxes with an edge length closest to 1× (small) and 1.5× (large) each individual’s hand aperture were selected for testing. If an individual’s hand aperture was the mean of two boxes, the smaller box was selected for testing. Four boxes (range: 3.6–7.0 cm) were used as the small box target, and seven boxes (range: 5.0–11.4 cm) were used as the large box target. A reflective marker was attached on top of each toy box to allow the Vicon system to capture the moving trajectory of the box.

During static trials, participants stood with feet side-by-side at the center of the walkway and their arms at their sides with fingers loosely closed (Figure 1B). The target box was set on top of a small tray adjusted to each participant’s hip height. The tray was positioned at one arm’s length from the participant in alignment with the midline of their body to allow individuals to bend forward to reach and pick up the target. After receiving the audio cue, participants reached and lifted the toy box using their right arm at their preferred speed. During dynamic trials, the tray was set at the midpoint of the walkway and on the right-hand side (Figure 1C). Participants started each trial by standing upright at the starting line of the walkway with their arms resting at their side and fingers relaxed. After receiving the audio cue, they walked on the walkway at their preferred speed to reach and lift the box from the tray and discontinued walking. The tray was positioned sideways to allow individuals to complete the trial without making a right turn or walking toward the right side of the walkway. During the task, participants were able to reach the box with their trunk facing forward and right arm slightly extended to the right. Participants completed three trials of reaching for each box (2: small vs. large) for each task condition (2: static vs. dynamic). All reaching conditions were administered as blocks, and the order of task conditions was randomized across individuals.

Upper limb kinematic data were low-pass filtered using a fourth order double pass Butterworth filter with a cutoff frequency of 7 Hz. Upper limb kinematics were the primary focus of the reaching tasks and were quantified by deriving the reaching duration, maximum reaching velocity, acceleration time, and reaching smoothness across the shoulder, elbow, and hand for both the small and large box conditions. The reaching onset was manually labeled by a trained scorer and double-checked by a separate trained scorer (Z.W.) based on the vertical displacement of the right-hand marker in Nexus (Vicon, Centennial, CO, USA). Reaching offset was defined as the first time point at which the velocity of the toy box marker exceeded 15% of its maximum in the vertical direction and continued for 0.2 s (i.e., 20 data points). Reaching duration was defined as the temporal duration between the reaching onset and offset. The maximum reaching velocity of each joint was then identified, based on the first derivative of the right shoulder, elbow, and hand markers from reaching onset to offset, respectively. The acceleration time was calculated for each joint and was defined as the duration from reaching onset until the maximum reaching velocity of the joint. To evaluate the smoothness of joint movement, the time-integrated, normalized, dimensionless squared jerk was derived for each joint based on the following formula [50,51]:

(1) $$Dimensionless\;Squared\;Jerk\;normalized\;by\;Peak\;Velocity\;\left(DSJP\right)\;=\;\sqrt{\int_{0}^{MT}\frac{J{\left(t\right)}^{2}}{2}dt\;\left(\frac{MT^{3}}{V_{peak}^{2}}\right)}$$

where *MT* represents the reaching duration, J(t) is the derivative of the acceleration, and *V_peak_* is the peak velocity. The *DSJP* was calculated for the right shoulder, elbow and hand.

### 2.3. Statistical Analyses

Statistical analyses were performed using IBM SPSS Statistics for Windows, version 25 (IBM Corp., Armonk, NY, USA). Between group comparisons (controls vs. FXTAS− vs. FXTAS+) were conducted using separate one-way ANOVAs for each dependent variable. For non-constrained walking, Bonferroni corrections were applied for three domains of gait measurements to limit type 1 errors, and the alpha level was adjusted to 0.02 (0.05/3 domains ≈ 0.02). For tests of reaching, the alpha level was set at 0.025 (0.05/2 reaching tasks = 0.025). Homogeneity of variance of all the gait and reaching variables was inspected using Levene’s test. Dependent variables which violated the assumption of normality were inspected and Log_10_ transformed.

Pearson correlations were conducted to determine relationships between the gait and reaching variables found to be significantly different between groups and age, full scale IQ, and CGG repeat lengths across all premutation carriers (i.e., FXTAS+, FXTAS−, and inconclusive individuals; N = 19). Due to the nominal rating of ICARS subscales and total score, Spearman’s rank correlation coefficient (rho) tests were used to examine relationships between the gait variables, the ICARS posture and gait subscale, and the total scores. Reaching variables were examined in relation to the ICARS kinetic function and the total scores. All correlations were interpreted as significant if *p* < 0.05 and |r| > 0.5.

## 3. Results

### 3.1. Participant Characteristics

The demographic, genetic, and clinical characteristics for each participant group (controls vs. FXTAS− vs. FXTAS+) are reported in Table 1, and individual characteristics are shown in Table 2. Participant groups showed no differences in height, weight, sex, or full scale IQ, although carriers with FXTAS were significantly older than the healthy controls.

### 3.2. Gait Performance in FXTAS

**Figure 2:**
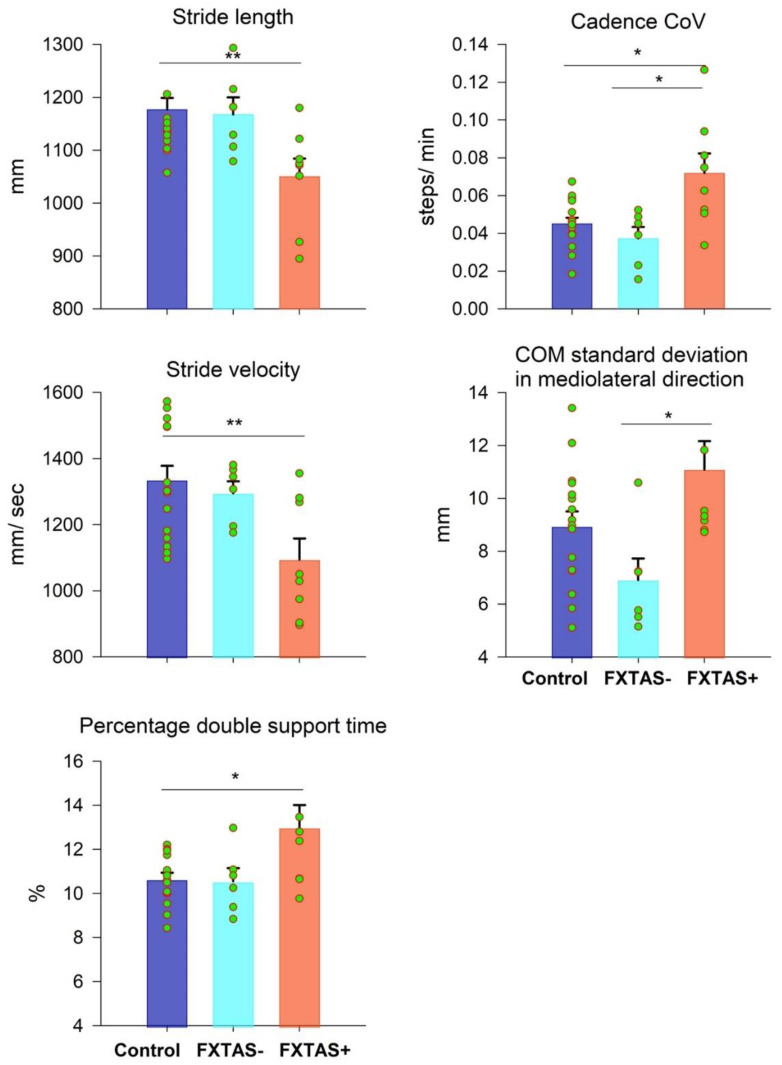
Mean differences in stride length, stride velocity, percentage of double support time, cadence coefficient of variation (CoV), and center of mass (COM) standard deviation in the mediolateral direction between the control (blue), FXTAS− (cyan), and FXTAS+ (orange) groups. Error bars represent standard errors. * *p* < 0.02. ** *p* < 0.01.

Figure 2 shows gait outcomes that were significantly different between participant groups (see Table S1 for descriptive statistics and the ANOVA results of all gait variables). FXTAS+ premutation carriers showed decreased stride lengths (group main effect: F_2,27_ = 5.825, *p* = 0.008; control−FXTAS+ = 126.49 mm, SE = 38.16 mm), decreased stride velocities (group main effect: F_2,27_ = 5.929, *p* = 0.007; control−FXTAS+ = 240.41 mm, SE = 70.69 mm), increased percentages of double support time (group main effect: F_2,27_ = 4.800, *p* = 0.016; control−FXTAS+ = −2.35%, SE = 0.775%), and an increased cadence CoV (group main effect: F_2,27_ = 5.926, *p* = 0.007; control−FXTAS+ = −0.0268 steps/min, SE = 0.0081 steps/min) compared with the healthy controls. They also showed an increased cadence CoV (group main effect: F_2,27_ = 7.48, *p* = 0.003; FXTAS−−FXTAS+ = −0.03465 steps/min, SE = 0.01 steps/min) and COM standard deviation in the mediolateral direction (group main effect: F_2,27_ = 5.096, *p* = 0.013; FXTAS−−FXTAS+ = −4.182 mm, SE = 1.32 mm) compared with the asymptomatic premutation carriers.

### 3.3. Reaching Performance in FXTAS

The participant groups did not differ on any reaching variables during static reaching (see Table S2 for descriptive statistics and ANOVA results).

**Figure 3:**
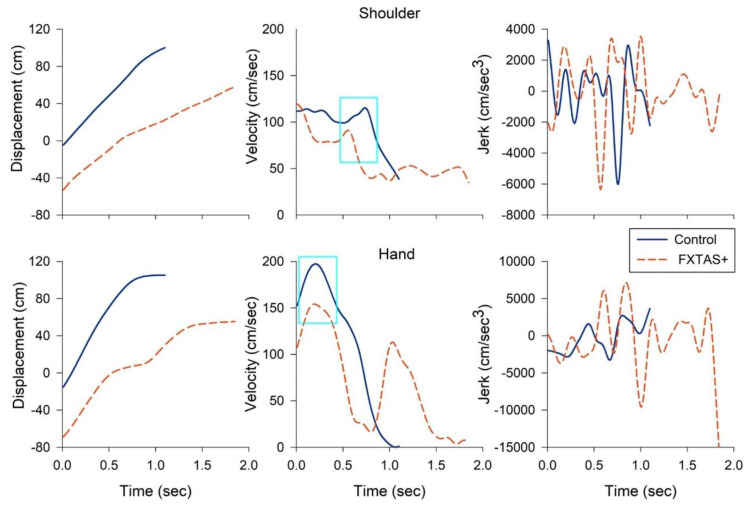
Representative kinematic profiles of the shoulder (**top**) and hand (**bottom**) of a 65-year-old patient with FXTAS (dashed orange line) and an age-matched healthy control (solid blue line) when they walked to reach and lift the small box. The FXTAS patient showed a prolonged reaching duration (**left**), decreased maximum reaching velocity of the shoulder and hand (**middle**, highlighted in a cyan box), and more oscillatory and jerkier joint movement (**right**) relative to the control participant. Time series were graphed from the reaching onset to offset. Jerk trajectories are shown in absolute values (cm/s^3^).

Figure 3 shows the representative displacement, velocity, and jerk time series of the right shoulder and hand of a 65-year-old FXTAS+ participant and an age-matched healthy control when they walked to reach the small box. The FXTAS+ participant showed a prolonged reaching duration, decreased maximum reaching velocity, and more oscillatory and jerkier joint movement relative to the control individual. Table 3 shows descriptive statistics and between-group comparisons of all the reaching variables during dynamic trials of walking-for-reaching. For the small box condition, carriers with FXTAS+ showed a decreased maximum reaching velocity (*p* = 0.001–0.015), prolonged acceleration time (*p* = 0.014–0.027), and jerkier movement (*p* = 0.004–0.015) of the right shoulder, elbow, and hand relative to the healthy controls. Additionally, FXTAS+ individuals showed jerkier hand movements compared with the carriers without FXTAS (*p* = 0.004). Carriers with FXTAS also showed increased reaching durations, relative to the controls and FXTAS− premutation carriers, although the group difference was not significant after adjusting for multiple comparisons (*p* = 0.029). For the large box condition, carriers with FXTAS+ showed decreased maximum velocities of the right shoulder and elbow (*p* = 0.001 and 0.019), prolonged acceleration times of all joints (*p* = 0.001 to 0.002), and jerkier hand movements (*p* = 0.008) relative to the healthy controls.

### 3.4. Demographic and Clinical Correlations

Increased age was associated with a decreased stride length (Table 4; r = −0.517, *p* = 0.028) and increased cadence CoV (r = 0.539, *p* = 0.021) in premutation carriers but not the controls. Neither IQ nor CGG repeat length were correlated with any gait measures in the premutation carriers. An increased IQ was associated with a reduced cadence CoV (r = −0.694, *p* = 0.003) and COM standard deviation (r = −0.501, *p* = 0.048) in the controls. The ICARS total and posture and gait ratings were correlated with all gait variables in the premutation carriers.

Neither IQ nor the CGG repeat length were correlated with any reaching variables in the premutation carriers (see Table S3 for detailed correlation results of the reaching variables with age, IQ, and CGG repeat length in the premutation carriers and healthy controls). Increased age was associated with decreased maximum shoulder velocity in the premutation carriers (r = −0.487, *p* = 0.035), though this effect did not reach our more stringent significance cutoffs (i.e., |r| > 0.5). Neither age nor IQ were correlated with any reaching kinematic variables in the healthy controls. Table 5 shows the correlations of the reaching variables with the ICARS kinetic function and total scores. For the small box condition, the ICARS kinetic ratings were associated with decreased maximum reaching velocities of all joints (*p* = 0.023–0.044) and jerkier movement of the hand (*p* = 0.034). The ICARS total score was associated with decreased maximum reaching velocity (*p* = 0.000–0.006) and movement smoothness (*p* = 0.027–0.033) of all joints, as well as increased shoulder acceleration time (*p* = 0.030) in the premutation carriers. For the large box condition, the ICARS kinetic ratings were associated with increased acceleration time of the right shoulder and hand (*p* = 0.010 and 0.028) and decreased maximum reaching velocity of the elbow and shoulder (*p* = 0.014 and 0.045), respectively. The ICARS total ratings were associated with increased shoulder, elbow, and hand acceleration times (*p* = 0.002–0.049), increased jerkier movement of the hand (*p* = 0.019), and decreased maximum reaching velocities of the shoulder and elbow (*p* = 0.000 and 0.003) in the premutation carriers.

## 4. Discussion

The current work aimed to (1) quantify the locomotor and upper limb kinematics during tasks of non-constrained walking and reaching in healthy controls and premutation carriers with and without FXTAS and (2) determine the relationships of gait and reaching variables with FXTAS symptoms and risk. To our knowledge, this is the first study to simultaneously examine the gait and upper limb kinematics in *FMR1* premutation carriers. Three key findings are highlighted. First, individuals with FXTAS showed multiple gait abnormalities compared with both the healthy controls and FXTAS− carriers, suggesting that assessment of a non-constrained gait could serve as a potential disease-specific behavioral marker to quantify and monitor FXTAS degeneration. Second, carriers with FXTAS also showed atypical reaching kinematics specific to conditions during which they were walking, indicating that gait alterations impacted their ability to perform activities of daily living, including upper limb actions. Third, gait and reaching abnormalities in FXTAS were associated with more severe clinical motor issues, indicating that quantitative assessments of gait and upper limb movement may reliably identify clinically relevant motor decline associated with FXTAS.

### 4.1. Gait Atypicalities in FXTAS

We found that individuals with FXTAS showed multiple quantifiable alterations of a non-constrained gait, including decreased stride length and stride velocity and increased percentage of double support time and variability of cadence and lateral movement of the COM. Atypicality of a non-constrained gait is a strong predictor of reduced quality of life and early mortality in healthy, aging individuals and individuals with neurological disorders [52,53]. Decreased gait amplitude and increased gait variability are associated with typical aging in healthy controls [54,55] and covary with cognitive ability (Table 4) [21,56,57]. However, more severe issues, as evident in the current study, in premutation carriers with FXTAS (Figure 2) appeared to be quantifiable, suggesting that these measurements may help differentiate disease processes from normative levels of decline in gait control.

Gait abnormalities, documented here in FXTAS+ individuals, implicate multiple sensorimotor brain regions supporting locomotor activities. The vermis and intermediate zone of the cerebellum are important sites involved in gait control. Circuits within the cerebellar vermis and intermediate zone are involved in integrating afferent inputs from central pattern generators, somatosensory receptors, and the interneurons of the spinocerebellar tracts to modulate spatial and temporal components of the gait at the periphery [58,59]. The lateral cerebellar circuits project to several cortical sensorimotor regions via the thalamus, including the primary motor and premotor cortices and the posterior parietal cortex, to facilitate the voluntary and adaptive control of walking [60]. Interruption to these cortical cerebellar pathways often results in gait ataxia, characterized by the inconsistent length, timing, and direction of steps, as well as increased and disoriented trunk or COM movement. Previous studies have documented reduced cerebellar volume associated with increased double support times and step widths in male *FMR1* premutation carriers relative to controls, implicating cortical cerebellar degeneration in aging *FMR1* premutation carriers [33]. Gait atypicalities, documented here in individuals with FXTAS, are quite similar to the impairments observed in cerebellar patients, including prolonged double support time and increased gait variability [61,62], suggesting that cerebellar disruptions contribute to gait decline in patients. These behavioral results are also in alignment with the neuropathology and anatomical MRI studies of patients with FXTAS, showing disrupted microstructural integrity of the cerebellar peduncles, inclusions in neurons and astrocytes of the cerebellum and brainstem, and general cerebral atrophy, suggesting that reduced cortical cerebellar modulation of locomotor coordination may serve as a subclinical indicator detectable prior to disease onset or early in the course of FXTAS degeneration [59,62,63,64,65].

We did not identify any differences in joint angular movement amongst the FXTAS+ or FXTAS− individuals relative to the controls. Several factors may account for this non-significant finding. First, the cortical cerebellar network modulates joint movements in a synergetic manner, during which a set of joints move with a coordinated timing and range of movement to achieve the task goal [66]. As such, it is possible that atypicalities of joint kinematics are reflected at the collective motor chain level, including the spatial and temporal components of the gait (Figure 2). Additionally, our joint angular measurements were derived at heel strikes and toe-offs alone, which do not afford quantification of possible deficits at other time points of a gait cycle in carriers with FXTAS. Furthermore, no *FMR1* premutation carriers in our sample reported any neurological concerns during their initial screening interview. This aspect of our study design allowed us to assess FXTAS during early or more mild stages, but also suggests that patients’ gait impairments may not have manifested yet at the joint level. Furthermore, musculoskeletal weakness (e.g., reductions in deep tendon reflexes and somatosensation) contributing to the control of lower limb joint movement has been reported primarily in male premutation carriers [1,35,36,37]. Our sample consisted of 68.4% female carriers, suggesting that neurodegeneration affecting joint movements may be specific to males with FXTAS [17,27]. It is also possible that female premutation carriers are less vulnerable to developing severe neurological impairments, including gait atypicalities, reflecting protective effects of the X chromosome [67,68]. Lastly, due to the heterogeneous presentations of FXTAS, there is a likelihood that not all carriers with FXTAS present aberrant joint angular movements during walking. Future studies involving larger cohorts of aging *FMR1* premutation carriers are warranted to examine the sensitivity of gait quantification for characterizing fundamental motor skill deficits and identifying subgroups of individuals with FXTAS who show predominant features of gait ataxia.

### 4.2. Reaching Atypicalities in FXTAS

We examined reaching during both standing and walking conditions to understand the contributions of previously documented postural [13,14,15] and gait alterations [17,27] to functional upper limb activities in FXTAS. Reaching abnormalities in FXTAS, including decreased maximum reaching velocity, prolonged acceleration time, and jerkier movement of the right arm, were specific to walking conditions, indicating that gait abnormalities likely contribute to difficulties executing goal-oriented reaching movements [25]. These findings indicate that assessment of the gait and gait-supported upper limb kinematics may be critical to identifying FXTAS and tracking aging processes among premutation carriers.

Target overshooting, increased variability of end point accuracy, and reduced multijoint coordination are the most prominent upper limb ataxic features in cerebellar patients [28,29,30,31,69]. Our findings of jerkier movement of the reaching joints in aging carriers with FXTAS (Figure 3 and Table 3) are consistent with upper limb ataxia, implicating the cortical cerebellar circuits involved in planning and executing smooth multijoint movements. Decreased maximum reaching velocities and prolonged acceleration times in individuals with FXTAS suggest non-cerebellar mechanisms are also disrupted. This hypothesis is supported by prior results from studies of manual control showing deficit patterns aligned with bradykinesia [12,70]. Specifically, we previously documented that aging premutation carriers showed prolonged reaction times, target undershooting, and reduced rates of initial force production during precision gripping [12]. Increased reaction times and movement times have also been documented in *FMR1* premutation carriers during standardized clinical assessments of manual dexterity [70]. These results suggest that upper limb atypicalities in aging individuals with FXTAS are characterized by slowed motor behavior, implicating the basal ganglia circuits [37,71]. This proposition is also supported by evidence of dopaminergic cell loss, the existence of Lewy bodies in surviving dopaminergic neurons in the substantia nigra [37,72,73], increased iron deposition in neuronal and glial cells of the putamen [74], and pre- and post-synaptic nigrostriatal dysfunction [75] in patients with FXTAS.

Although reaching kinematics appeared intact in carriers with FXTAS during reaching-in-standing (Table S2), we would not rule out the possibility that compromised cerebellar and basal ganglia circuits may still affect upper limb control while standing. For the small box condition, both the reaching duration (*p* = 0.032, Cohen’s d = 0.475) and acceleration time of the shoulder joint (*p* = 0.045, Cohen’s d = 0.673) showed medium to large effect sizes. Additionally, the maximum velocity of the elbow (*p* = 0.051, Cohen’s d = 0.430) and acceleration time of the elbow (*p* = 0.051, Cohen’s d = 0.422) and hand (*p* = 0.051, Cohen’s d = 0.418) also showed medium effect sizes during the large box condition, suggesting that FXTAS may involve more subtle or variable reductions in reaching movement speed that also are present during standing.

### 4.3. Upper and Lower Limb Kinematics and Their Relation to Demographic and Clinical Characteristics in FMR1 Premutation Carriers

Consistent with previous studies [17,26,27,33], we found that gait abnormalities in premutation carriers were strongly correlated with the ICARS posture and gait subscores and total scores, suggesting that kinematic assessment of non-constrained walking may reliably identify clinically relevant declines in FXTAS. In contrast, during walking-for-reaching trials, the ICARS kinetic subscores did not correlate with the acceleration time or jerk measures in the premutation carriers. This finding might be accounted for by several factors. First, it is possible that upper limb movement impairments represent distinct neurodegenerative mechanisms that are different from degeneration specific to ataxia and assessed by the ICARS. Second, it is possible that clinical rating scales are less sensitive than our quantitative approaches for identifying subtle or early indicators of FXTAS, particularly from a subclinical sample of *FMR1* premutation carriers.

Increased age was associated with decreased stride length and increased cadence CoV in the *FMR1* premutation carriers, while age was not associated with any gait measurements in the healthy controls. This finding demonstrates that premutation carriers may show an earlier or more rapid decline in gait control relative to healthy aging [3,4,76,77], though it should be noted that our control group was younger than our *FMR1 *premutation carriers (Table 1). We also found that lower IQ was associated with an increased cadence CoV and COM standard deviation in the healthy controls, but not in the *FMR1* premutation carriers, suggesting that sensorimotor and broader cognitive issues may be independently affected during aging in premutation carriers. Furthermore, the CGG repeat length was not associated with any gait or reaching variables in the *FMR1* premutation carriers (Table 4 and Table S3). Shichman et al. [70] documented a possible inverted U shape between visual memory performance and CGG repeat size in *FMR1* premutation carriers, postulating increased rates of memory errors in carriers with mid-length expansions. It is possible that CGG length and FXTAS symptoms and risk vary in a non-linear fashion. Future studies involving larger cohorts of aging *FMR1* premutation carriers across a broad range of CGG repeat lengths are warranted to elucidate patterns of potential non-linear relationships and better understand molecular mechanisms of different sensorimotor issues in FXTAS.

## 5. Limitations and Future Directions

Our results should be considered in the context of a few limitations. First, the study consisted of a small cohort of *FMR1* premutation carriers that limited our statistical power and ability to capture variability in clinical and motor presentations among carriers. Future studies involving larger cohorts of premutation carriers with and without FXTAS are needed to examine the sensitivity of gait and reaching variables for characterizing fundamental motor skill deficits and their variation across individuals and disease stages. Second, our sample primarily consisted of female premutation carriers. Given that FXTAS penetrance is reduced in females, our results might be biased against identifying movement alterations that may be more prominent in male premutation carriers. Third, while reporting behavioral findings related to the CGG repeat length is important, other molecular measurements, including mRNA, methylation ratios, and FMR protein will be important for clarifying the molecular mechanisms of FXTAS onset, risk, and progression. Lastly, these tasks were administered in the context of a larger assessment battery. Given the time constraints and concerns regarding participant fatigue, three trials were administered for each of the gait and reaching tasks. Although our findings on non-constrained walking were consistent with previous work [17,26,27,33], future studies shall include more trials to increase the reliability of the kinematic quantifications for gait and reaching [78].

## 6. Conclusions

Individuals with FXTAS showed gait alterations relative to healthy controls and asymptomatic *FMR1* premutation carriers. Carriers with FXTAS also showed atypical upper limb kinematics, presenting features similar to basal ganglia-related bradykinesia and Parkinsonism. These findings highlight the critical need to develop targeted and objective measurement strategies to distinguish FXTAS and asymptomatic premutation carriers, as well as the ataxic and bradykinetic features of FXTAS.

## List of Abbreviations

FXTASFragile X-associated tremor/ataxia syndromeCGGCytosine-guanine-guaninemRNAMessenger RNAMRIMagnetic resonance imagingICARSInternational Cooperative Ataxia Rating ScaleSB-5Stanford–Binet Intelligence Scales, Fifth EditionDSJPDimensionless squared jerk normalized by peak velocityCoVCoefficient of variation

## Figures and Tables

**Table 1:** Demographics and clinical characteristics of healthy controls and premutation carriers with and without fragile X-associated tremor/ataxia syndrome (FXTAS).

Characteristics	Controls (*n* = 16)	FXTAS− (*n* = 6)	FXTAS+ (*n* = 9)	*F*	*p*
Age (yr)	53.13 (8.41)	57.00 (6.20)	63.33 (8.35) ^#^	**4.645**	0.018 *
Height (cm)	167.61 (7.77)	167.38 (12.76)	165.14 (5.09)	0.269	0.766
Leg length (cm)	86.31 (7.21)	89.33 (5.78)	85.93 (2.61)	0.686	0.512
Weight (kg)	80.77 (16.41)	82.56 (26.42)	83.77 (25.11)	0.060	0.941
Male (*n*) ^φ^	8	2	3	0.883	0.643
Full scale IQ	106.63 (14.15)	99.50 (8.14)	102.67 (16.73)	0.622	0.544
CGG repeats	—	74 (60–102)	83 (58-107)	0.416	0.531
ICARS speech	—	0 (0)	0 (0–2)	1.493	0.245
ICARS kinetic	—	0 (0)	2 (1–7) ^†^	**9.288**	0.010 *
ICARS oculomotor	—	0.5 (0–1)	0 (0–3)	0.468	0.507
ICARS posture and gait	—	1.5 (0–3)	5 (1–7) ^†^	**12.039**	0.005 **
ICARS total	—	2 (0–4)	8 (2–19) ^†^	**9.510**	0.009 **

Age, height, leg length, and weight are reported as (mean (SD)); CGG repeats and ICARS scores are reported as (median (range)). ^φ^ Chi-square statistics. ^#^ FXTAS+ group differs from healthy controls. ^†^ FXTAS+ group differs from FXTAS− group. * *p* < 0.05, ** *p* < 0.01. Bold highlight significant findings.

**Table 2:** Detailed demographic, genetic, and clinical characteristics for each *FMR1* gene premutation carrier.

ID	Age	Sex	CGG	ICARS					T2 Scan	Neurological Exam	Clinical Classification
				Speech	Kinetic	Oculomotor	Gait Posture	Total			
1	55	F	87	0	0	1	1	2	Generalized white matter lesion, cerebral atrophy type 1	Tremor (−) Gait ataxia (−)	FXTAS−
2	61	F	102	0	0	1	2	3	Generalized white matter lesion, cerebral atrophy type 2	Tremor (−) Gait ataxia (−)	FXTAS−
3	58	M	60	0	0	1	3	4	(−)	Tremor (−) Gait ataxia (−)	FXTAS−
4	58	M	63	0	0	0	2	2	(−)	Tremor (+) Gait ataxia (−)	FXTAS−
5	46	F	68	0	0	0	0	0	Mild white matter lesion, white matter hypersensitivity	Tremor (−) Gait ataxia (−)	FXTAS−
6	64	F	80	0	0	0	1	1	Mild white matter lesion, white matter hypersensitivity, cerebral atrophy type 2	Tremor (−) Gait ataxia (−)	FXTAS−
7	54	F	99	0	2	0	5	7	Mild cerebellar features	Tremor (+): mild	Probable
8	59	F	107	0	1	2	5	8			Probable
9	71	M	85	1	2	2	7	12	Generalized white matter lesion, cerebral atrophy type 3	Tremor (+) Gait ataxia (+)	Definite
10	52	F	81	0	1	0	4	5	Cerebral atrophy type 1	Tremor (+) Gait ataxia (+)	Probable
11	77	F	75	2	7	3	7	19		Tremor (+) Gait ataxia (+)	Probable
12	67	F	62	0	1	0	1	2	Mild white matter lesion, white matter hypersensitivity	Tremor (+) Gait ataxia (−)	Possible
13	65	M	58	0	3	0	5	8	(−)	Tremor (+) Gait ataxia (+)	Probable
14	57	M	93	0	5	0	3	8	Suspected middle cerebellar peduncle sign, cerebral atrophy type 1, fourth ventricle widening, atrophy of cerebellum and brainstem	Tremor (+) Gait ataxia (−)	Possible
15	68	F							(−)	Tremor (+) Gait ataxia (+)	Probable
16	62	F	102	0	0	0	0	0			Inconclusive
17	70	F	90	0	2	2	2	6		Tremor (+) Gait ataxia (−)	Inconclusive
18	61	M	64								Inconclusive
19	59	F	78						Mild white matter lesion, white matter hypersensitivity		Inconclusive

(−) entry: no abnormality was identified. No entry: data was not collected.

**Table 3:** Reaching outcomes (mean (SD)) during dynamic walking trials for the healthy controls and premutation carriers with and without FXTAS.

	Control (*n* = 16)	FXTAS− (*n* = 6)	FXTAS+ (*n* = 9)	*F*	*p*
**Small box condition**					
Reaching duration (s)	1.25 (0.33)	1.35 (0.34)	1.67 (0.40)	4.047	0.029
Hand max velocity (cm/s)	214.64 (45.18)	203.57 (36.57)	163.48 (29.08) ^#^	**4.872**	0.015 *
Elbow max velocity (cm/s)	147.50 (14.38)	138.62 (17.49)	115.54 (22.46) ^#^	**8.991**	0.001 **
Shoulder max velocity (cm/s)	119.36 (11.69)	114.83 (11.33)	97.03 (19.92) ^#^	**6.999**	0.003 **
Hand acceleration time (s) ^φ^	1.65 (0.32)	1.82 (0.28)	2.33 (0.83) ^#^	**4.974**	0.014 *
Elbow acceleration time (s) ^φ^	1.64 (0.37)	1.79 (0.31)	2.32 (0.88) ^#^	**4.381**	0.022 *
Shoulder acceleration time (s) ^φ^	1.57 (0.36)	1.70 (0.33)	2.26 (0.93) ^#^	**4.120**	0.027 *
Hand DSJP ^φ^	29.88 (10.21)	34.49 (12.42)	54.27 (18.85) ^†^	**6.829**	0.004 **
Elbow DSJP ^φ^	31.20 (14.00)	33.46 (11.76)	56.86 (24.89) ^#^	**5.242**	0.012 *
Shoulder DSJP ^φ^	33.75 (15.44)	35.11 (11.18)	60.22 (26.87) ^#^	**4.932**	0.015 *
**Large box condition**					
Reaching duration (s)	1.28 (0.33)	1.48 (0.21)	1.63 (0.44)	3.124	0.060
Hand max velocity (cm/s)	208.18 (43.89)	212.05 (43.00)	165.04 (31.73)	3.801	0.035
Elbow max velocity (cm/s)	145.93 (16.63)	138.40 (1.683)	115.83 (19.12) ^#^	**8.697**	0.001 **
Shoulder max velocity (cm/s)	116.01 (13.71)	117.59 (12.19)	98.92 (17.40) ^#^	**4.606**	0.019 *
Hand acceleration time (s)	1.68 (0.42)	1.77 (0.27)	2.46 (0.57) ^†^	**8.962**	0.001 **
Elbow acceleration time (s)	1.72 (0.43)	1.73 (0.28)	2.43 (0.58) ^†^	**7.583**	0.002 **
Shoulder acceleration time (s)	1.61 (0.42)	1.65 (0.28)	2.37 (0.67) ^†^	**7.687**	0.002 **
Hand DSJP ^φ^	30.25 (9.54)	37.26 (7.51)	51.45 (21.53) ^#^	**5.692**	0.008 **
Elbow DSJP	32.62 (15.35)	40.17 (9.67)	52.43 (29.28)	2.916	0.071
Shoulder DSJP	35.00 (16.41)	40.66 (7.86)	56.89 (29.19)	3.429	0.044

DSJP = dimensionless squared jerk normalized by peak velocity (unitless). ^φ^ Log_10_ transformed variables. # FXTAS+ group differs from healthy controls. † FXTAS+ group differs from FXTAS− group. * *p* < 0.025. ** *p* < 0.01. Bold highlight significant findings.

**Table 4:** Correlations between gait features and demographic and clinical characteristics for premutation carriers and controls.

	Age	IQ	CGG Repeats	ICARS Posture Gait	ICARS Total
*FMR1* premutation carriers (*n* = 19)					
Stride length (mm)	r = −0.517, *p* = 0.028 *	r = −0.206, *p* = 0.411	r = −0.225, *p* = 0.386	rho = −0.689, *p* = 0.009 **	rho = 0.609, *p* = 0.027 *
Stride velocity (mm/s)	r = −0.286, *p* = 0.250	r = −0.248, *p* = 0.321	r = −0.143, *p* = 0.585	rho = −0.697, *p* = 0.008 **	rho = −0.575, *p* = 0.040 *
Pct. double support time (%)	r = 0.126, *p* = 0.619	r = 0.246, *p* = 0.325	r = 0.288, *p* = 0.262	rho = 0.667, *p* = 0.013 *	rho = 0.559, *p* = 0.047 *
Cadence CoV (steps/min)	r = 0.539, *p* = 0.021 *	r = 0.306, *p* = 0.217	r = 0.161, *p* = 0.538	rho = 0.761, *p* = 0.003 **	rho = 0.672, *p* = 0.012 *
Step COM SD (mm)	r = 0.179, *p* = 0.477	r = 0.143, *p* = 0.595	r = −0.006, *p* = 0.982	rho = 0.644, *p* = 0.017 *	rho = 0.573, *p* = 0.041 *
Controls (*n* = 16)					
Stride length (mm)	r = 0.145, *p* = 0.592	r = 0.456, *p* = 0.076	—	—	—
Stride velocity (mm/s)	r = 0.075, *p* = 0.783	r = 0.461, *p* = 0.072	—	—	—
Pct. double support time (%)	r = 0.156, *p* = 0.565	r = −0.046, *p* = 0.865	—	—	—
Cadence CoV (steps/min)	r = 0.035, *p* = 0.897	r = −0.694, *p* = 0.003 **	—	—	—
Step COM SD (mm)	r = −0.033, *p* = 0.902	r = −0.501, *p* = 0.048 *	—	—	—

Pct. double support time = percentage of double support time; Step COM SD = COM standard deviation in the mediolateral direction. * *p* < 0.05. ** *p* < 0.01. Bold highlight significant findings.

**Table 5:** Correlation between reaching and clinical characteristics for the premutation carriers.

	ICARS Kinetic	ICARS Total
Small box condition		
Hand max velocity	rho = −0.510, *p* = 0.044 *	rho = −0.651, *p* = 0.006 **
Elbow max velocity	rho = −0.535, *p* = 0.033 *	rho = −0.756, *p* = 0.001 **
Shoulder max velocity	rho = −0.562, *p* = 0.023 *	rho = −0.816, *p* = 0.000 **
Hand acceleration time	rho = 0.244, *p* = 0.362	rho = 0.417, *p* = 0.108
Elbow acceleration time	rho = 0.253, *p* = 0.344	rho = 0.400, *p* = 0.125
Shoulder acceleration time	rho = 0.343, *p* = 0.193	rho = 0.542, *p* = 0.030 *
Hand DSJP	rho = 0.532, *p* = 0.034 *	rho = 0.534, *p* = 0.033 *
Elbow DSJP	rho = 0.476, *p* = 0.062	rho = 0.551, *p* = 0.027 *
Shoulder DSJP	rho = 0.417, *p* = 0.108	rho = 0.552, *p* = 0.027 *
Large box condition		
Elbow max velocity	rho = −0.599, *p* = 0.014 *	rho = −0.783, *p* = 0.000 **
Shoulder max velocity	rho = −0.507, *p*=.045 *	rho = −0.691, *p* = 0.003 **
Hand acceleration time	rho = 0.547, *p*= 0.028 *	rho = 0.632, *p* = 0.009 **
Elbow acceleration time	rho = 0.454, *p* = 0.077	rho = 0.499, *p* = 0.049 *
Shoulder acceleration time	rho = 0.624, *p* = 0.010 **	rho = 0.709, *p* = 0.002 **
Hand DSJP	rho = 0.368, *p* = 0.161	rho = 0.577, *p* = 0.019 *

* *p* < 0.05. ** *p* < 0.01. Bold highlight significant findings.

## Data Availability

The data presented in this study are available on request from the corresponding author. The data are not publicly available due to privacy/ ethical restrictions (e.g., heir containing information that could compromise the privacy of research participants).

## Supplementary Materials

The following are available online at https://www.mdpi.com/2076-3425/11/1/13/s1. Table S1. Descriptive statistics [mean (SD)] and between group comparisons of all gait variables during non-constrained walking trials. Table S2. Descriptive statistics [mean (SD)] and between group comparisons of all reaching variables during reaching-in-standing trials. Table S3. Correlation coefficients of reaching variables during walking-for-reaching trials with demographic and clinical characteristics for healthy controls and premutation carriers.
