# Supplementary material for: Upper and Lower Limb Movement Kinematics in Aging FMR1 Gene Premutation Carriers
Source: Brain Sci. 2020 Dec 24;11(1):13. doi: 10.3390/brainsci11010013 (PMC7823457; doi:10.3390/brainsci11010013)
Supplement: Supplementary file 1 [file brainsci-11-00013-s001.pdf]

Table S1. Descriptive statistics [mean (SD)] and between group comparisons of all gait variables during non-constrained walking trials. Dependent variables (bold-faced) that showed significant group main effect at the alpha level of .02 are depicted in Figure 2 of the article. Stride time CoV was significant at the alpha level of 0.05.

Gait Variables	Control (*n*=18)	FXTAS− (*n*=6)	FXTAS+ (*n*=9)	*F*	*p*
Spatial domain					
Stride length (mm)	1176.96 (87.48)	1167.54 (79.21)	1050.48 (95.24)	5.825[#]	0.008[**]
Stride length CoV (mm)	0.0482 (0.0284)	0.0488 (0.0252)	0.0561 (0.0277)	0.234	0.793
Step width (mm)	73.32 (30.76)	68.89 (24.16)	78.28 (35.06)	0.162	0.851
Step width CoV (mm)	0.6254 (0.7163)	0.3795 (0.1366)	0.6187 (0.4119)	0.429	0.656
Temporal domain					
Stride velocity (mm/s)	1334.96 (173.32)	1295.08 (88.67)	1094.55 (181.14)	5.929[#]	0.007[**]
Stride velocity CoV (mm/s)	0.0555 (0.0339)	0.0561 (0.0286)	0.0638 (0.0253)	0.204	0.817
Pct. double support time (%)[ϕ]	10.65 (1.13)	10.55 (1.46)	13.00 (2.85)	4.800[#]	0.016[*]
Pct. Double support time CoV (%)	0.1425 (0.0731)	0.1425 (0.0808)	0.1509 (0.0593)	0.041	0.960
Stride time (s)	0.8912 (0.0860)	0.9040 (0.0590)	0.9759 (0.1263)	2.214	0.129
Stride time CoV (s)	0.0322 (0.0141)	0.0288 (0.0150)	0.0486 (0.0202)	3.530	0.043
Cadence (steps/min)	135.68 (12.98)	133.21 (8.49)	125.14 (15.16)	1.794	0.186
Cadence CoV (steps/min)[ϕ]	0.0452 (0.0124)	0.0373 (0.0148)	0.0720 (0.0292)	5.926[#,†]	0.007[**]
Kinematic domain					
COM standard deviation (mm)	8.94 (2.26)	6.91 (2.01)	11.09 (3.04)	5.096[†]	0.013[*]
Heel strike ipsilateral knee angle (deg)	3.56 (5.72)	4.50 (7.06)	2.38 (5.51)	0.225	0.800
Heel strike ipsilateral knee angle CoV (deg)	0.5778 (3.1355)	0.3743 (0.3559)	4.43 (11.62)	1.111	0.344
Heel strike contralateral knee angle (deg)	7.11	8.81	4.39	0.983	0.387

	(7.05)	(3.53)	(5.15)		
Heel strike contralateral knee angle CoV (deg)	1.56 (2.88)	0.3114 (0.1586)	0.7957 (2.08)	0.678	0.516
Heel strike ipsilateral ankle angle (deg)	−1.83 (16.82)	0.5732 (10.91)	−7.64 (25.32)	0.392	0.680
Heel strike ipsilateral ankle angle CoV (deg)	0.9405 (7.42)	0.4239 (.8909)	−1.60 (5.41)	0.456	0.638
Heel strike contralateral ankle angle (deg)	11.06 (28.25)	13.73 (7.65)	19.95 (3.22)	0.462	0.635
Heel strike contralateral ankle angle CoV (deg)	0.1202 (0.6449)	66.09 (1623.80)	1.12 (2.28)	2.185	0.135
Toe off ipsilateral knee angle (deg)	29.84 (10.28)	32.05 (7.34)	25.95 (11.03)	0.697	0.507
Toe off ipsilateral knee angle CoV (deg)	0.3133 (0.3223)	0.1388 (0.0590)	0.3106 (.2972)	0.890	0.422
Toe off contralateral knee angle (deg)	11.10 (5.14)	10.86 (6.99)	6.10 (7.01)	1.965	0.160
Toe off contralateral knee angle CoV (deg)	0.5919 (0.5591)	0.4300 (0.2469)	−0.0028 (2.51)	0.519	0.601
Toe off ipsilateral ankle angle (deg)	−10.30 (15.16)	−9.03 (13.48)	−9.01 (11.29)	0.031	0.969
Toe off ipsilateral ankle angle CoV (deg)	−0.1987 (22.82)	−2.65 (2.94)	−1.43 (2.10)	1.953	0.161
Toe off contralateral ankle angle (deg)	−1.84 (19.05)	0.7962 (8.00)	−0.7495 (6.57)	0.069	0.933
Toe off contralateral ankle angle CoV(deg)	−.2727 (8.86)	0.8777 (1.62)	−0.3312 (4.00)	0.068	0.934

CoV=coefficient of variation; $^{\phi}$ Log$_{10}$ transformed variables; $^{\#}$ FXTAS+ group differ from healthy controls; † FXTAS+ group differ from FXTAS− group; $^{*}p<.02$, $^{**}p<.01$.

Table S2. Descriptive statistics [mean (SD)]and between group comparisons of all reaching variables during reaching−in−standing trials. In general, no reaching variable has shown a significant group main effect based on adjusted alpha at 0.025 (See method). Reaching duration and shoulder acceleration time of the small box condition were significant at the alpha level of 0.05.

	**Control (*n*=16)**	**FXTAS− (*n*=6)**	**FXTAS+ (*n*=9)**	***F***	***p***
**Small box condition**					
Reaching duration (s)	1.14 (0.19)	1.39 (0.26)	1.28 (0.17)	**3.910**	**0.032***
Hand max velocity (cm/s)	101.76 (21.91)	95.76 (22.48)	90.13 (14.45)	0.977	0.389
Elbow max velocity (cm/s)	87.01 (16.47)	73.60 (23.40)	70.46 (10.97)	3.324	0.051
Shoulder max velocity (cm/s)	34.89 (13.35)	26.07 (11.92)	31.45 (16.04)	0.891	0.422
Hand acceleration time (s)	3.14 (0.77)	1.78 (1.40)	2.10 (1.87)	3.318	0.051
Elbow acceleration time (s)	3.30 (0.78)	1.92 (1.34)	2.29 (1.87)	3.324	0.051
Shoulder acceleration time (s)	3.46 (0.77)	2.13 (1.35)	2.39 (1.81)	**3.466**	**0.045***
Hand DSJP$^{\phi}$	33.60 (6.02)	39.90 (12.88)	36.62 (11.56)	0.679	0.515
Elbow DSJP	25.50 (6.74)	35.64 (17.82)	33.86 (25.36)	1.226	0.309
Shoulder DSJP	23.29 (13.25)	36.58 (27.67)	25.76 (8.73)	1.543	0.231
**Large box condition**					
Reaching duration (s)	1.17 (0.21)	1.30 (0.31)	1.32 (0.19)	1.405	0.263.
Hand max velocity (cm/s)	101.09 (27.69)	91.64 (14.41)	87.26 (20.41)	0.915	0.413
Elbow max velocity (cm/s)	81.70 (17.30)	80.69 (21.29)	73.72 (19.21)	0.512	0.605
Shoulder max velocity (cm/s)	35.24 (12.50)	28.12 (9.90)	29.04 (6.71)	1.324	0.284
Hand acceleration time (s)	3.32 (0.69)	2.34 (1.15)	2.64 (2.18)	1.236	0.308
Elbow acceleration time (s)	3.47 (0.73)	2.11 (1.33)	2.79 (2.15)	2.148	0.137
Shoulder acceleration time (s)	3.63 (0.70)	2.31 (1.35)	2.90 (2.12)	2.143	0.138
Hand DSJP	34.96 (10.52)	37.28 (13.31)	38.99 (8.23)	0.420	0.662
Elbow DSJP	29.12 (13.32)	30.59 (15.79)	30.10 (10.54)	0.031	0.970
Shoulder DSJP	24.21 (11.86)	28.16 (13.90)	24.61 (8.86)	0.231	0.796

*$p<0.05$; $^{\phi}$ Log_{10} transformed variables;

Table S3. Correlation coefficients of reaching variables during walking–for–reaching trials with demographic and clinical characteristics for healthy controls and premutation carriers (including FXTAS–, FXTAS+ and inconclusive individuals).

**Small box condition**	**Age**	**IQ**	**CGG repeats**
FMR1 premutation carriers (n=19)			
Hand max velocity (cm/s)	r= –0.108, p=0.667	r= –0.052, p=0.833	r=0.100, p=0.692
Elbow max velocity (cm/s)	r= –0.228, p=0.347	r= –0.142, p=0.561	r= –0.146, p=0.563
Shoulder max velocity (cm/s)	r= –0.332, p=0.165	r= –0.222, p=0.360	r=–0.304, p=0.220
Hand acceleration time (s)	r=0.253, p=0.295	r=0.278, p=0.250	r=0.208, p=0.408
Elbow acceleration time (s)	r=0.246, p=0.309	r=0.255, p=0.293	r=0.237, p=0.344
Shoulder acceleration time (s)	r=0.326, p=0.173	r=0.262, p–0.278	r=0.218, p=0.385
Hand DSJP	r=0.183, p=0.453	r=0.088, p=0.721	r= –0.037, p=0.884
Elbow DSJP	r=0.126, p=0.607	r=0.200, p=0.413	r=0.052, p=0.837
Shoulder DSJP	r=0.157, p=0.522	r=0.200, p=0.412	r=0.194, p=0.440
Controls (n=16)			
Hand max velocity (cm/s)	r= –0.256, p=0.338	r= –0.220, p=0.412	—
Elbow max velocity (cm/s)	r=0.023, p=0.931	r= –0.131, p=0.630	—
Shoulder max velocity (cm/s)	r=0.189, p=0.482	r=0.242, p=0.367	—
Hand acceleration time (s)	r= –0.023, p=0.932	r= –0.188, p=0.486	—
Elbow acceleration time (s)	r= –0.012, p=0.964	r= –0.194, p=0.472	—
Shoulder acceleration time (s)	r= –0.105, p=0.699	r= –0.261, p=0.329	—
Hand DSJP	r= –0.124, p=0.648	r= –0.169, p=0.533	—
Elbow DSJP	r=0.041, p=0.881	r= –0.129, p=0.633	—
Shoulder DSJP	r= –0.035, p=0.899	r= –0.199, p=0.460	—

**Large box condition**	**Age**	**IQ**	**CGG repeats**
FMR1 premutation carriers (n=19)			
Hand max velocity (cm/s)	r= –0.198, p=0.416	r= –0.266, p=0.351	r=0.130, p=0.608
Elbow max velocity (cm/s)	r= –0.299, p=0.214	r= –0.329, p=0.170	r= –0.135, p=0.592
Shoulder max velocity (cm/s)	**r= –0.487, p=0.035***	r= –0.423, p=0.071	r= –0.198, p=0.430
Hand acceleration time (s)	r=0.217, p=0.372	r=0.269, p=0.265	r=0.071, p=0.779
Elbow acceleration time (s)	r=0.234, p=0.335	r=0.301, p=0.210	r=0.086, p=0.734
Shoulder acceleration time (s)	r=0.271, p=0.262	r=0.184, p=0.452	r=0.108, p=0.669
Hand DSJP	r=0.161, p=0.511	r=0.244, p=0.314	r=0.161, p=0.524
Elbow DSJP	r=0.200, p=0.411	r=0.298, p=0.215	r=0.104, p=0.680
Shoulder DSJP	r=0.235, p=0.333	r=0.285, p=0.237	r=0.046, p=0.857

Controls (n=16)			
Hand max velocity (cm/s)	r= −0.167, p=0.537	r= −0.030, p=0.912	—
Elbow max velocity (cm/s)	r=0.142, p=0.599	r=0.054, p=0.843	—
Shoulder max velocity (cm/s)	r=0.228, p=0.396	r=0.309, p=0.244	—
Hand acceleration time (s)	r= −0.046, p=0.866	r= −0.388, p=0.138	—
Elbow acceleration time (s)	r=0.012, p=0.966	r= −0.363, p=0.168	—
Shoulder acceleration time (s)	r= −0.093, p=0.731	r= −0.466, p=0.069	—
Hand DSJP	r=0.033, p=0.902	r= −0.041, p=0.881	—
Elbow DSJP	r=0.095, p=0.727	r=0.026, p=0.924	—
Shoulder DSJP	r=0.104, p=0.702	r= −0.032, p=0.908	—

$^{*}p<0.05$.
